# Theoretical Analysis of the Performance of Glucose Sensors with Layer-by-Layer Assembled Outer Membranes

**DOI:** 10.3390/s121013402

**Published:** 2012-10-01

**Authors:** Robert A. Croce, Santhisagar Vaddiraju, Fotios Papadimitrakopoulos, Faquir C. Jain

**Affiliations:** 1 Electrical and Computer Engineering, University of Connecticut, 371 Fairfield Way, Storrs, CT 06269, USA; E-Mail: rjc03001@engr.uconn.edu; 2 Biorasis Inc., Technology Incubation Program, University of Connecticut, Storrs, CT 06269, USA; 3 Nanomaterials Optoelectronics Laboratory, Polymer Program, Institute of Materials Science, University of Connecticut, Storrs, CT 06269, USA; E-Mails: sagar@bio-orasis.com (S.V.); papadim@ims.uconn.edu (F.P.); 4 Department of Chemistry, University of Connecticut, Storrs, CT 06269, USA

**Keywords:** amperometric glucose sensors, mathematical modeling, biosensors, Michaelis-Menten constant, enzyme kinetics, layer-by-layer assembly, polymer chemistry, finite difference schemes, differential equations, analyte diffusion

## Abstract

The performance of implantable electrochemical glucose sensors is highly dependent on the flux-limiting (glucose, H_2_O_2_, O_2_) properties of their outer membranes. A careful understanding of the diffusion profiles of the participating species throughout the sensor architecture (enzyme and membrane layer) plays a crucial role in designing a robust sensor for both *in vitro* and *in vivo* operation. This paper reports the results from the mathematical modeling of Clark's first generation amperometric glucose sensor coated with layer-by-layer assembled outer membranes in order to obtain and compare the diffusion profiles of various participating species and their effect on sensor performance. Devices coated with highly glucose permeable (HAs/Fe^3+^) membranes were compared with devices coated with PSS/PDDA membranes, which have an order of magnitude lower permeability. The simulation showed that the low glucose permeable membrane (PSS/PDDA) sensors exhibited a 27% higher amperometric response than the high glucose permeable (HAs/Fe^3+^) sensors. Upon closer inspection of H_2_O_2_ diffusion profiles, this non-typical higher response from PSS/PDDA is not due to either a larger glucose flux or comparatively larger O_2_ concentrations within the sensor geometry, but rather is attributed to a 48% higher H_2_O_2_ concentration in the glucose oxidase enzyme layer of PSS/PDDA coated sensors as compared to HAs/Fe^3+^ coated ones. These simulated results corroborate our experimental findings reported previously. The high concentration of H_2_O_2_ in the PSS/PDDA coated sensors is due to the low permeability of H_2_O_2_ through the PSS/PDDA membrane, which also led to an undesired increase in sensor response time. Additionally, it was found that this phenomenon occurs for all enzyme thicknesses investigated (15, 20 and 25 nm), signifying the need for a holistic approach in designing outer membranes for amperometric biosensors.

## 1. Introduction

The design and implementation of miniaturized implantable potentiometric or amperometric sensors for glucose monitoring has recently gained considerable attention in light of their ability to be integrated with integrated electronic circuits in the development of continuous glucose monitoring systems [1–6]. Among these, Clark's type amperometric sensors drew a lot of attention which is owed to their design simplicity and ease of fabrication. First generation Clark-based amperometric sensors employ a glucose oxidase (GO_x_) enzyme immobilized on top of the working electrode [7]. The flavin adenine dinucleotide (FAD) redox cofactor of GO_x_ catalyzes the oxidation of glucose to glucarolactone, as shown in Equations (1) and (2):

(1) $$\text{Glucose}+GO_{x}(\text{FAD})\to \text{Glucolactone}+GO_{x}(\text{FAD}H_{2})$$

(2) $$GO_{x}(\text{FAD}H_{2})+O_{2}\to GO_{x}(\text{FAD})+H_{2}O_{2}.$$

The generated H_2_O_2_ is amperometrically assessed on the surface of working electrode by the application of the appropriate redox potential, hence relating the current to glucose concentration,

(3) $$H_{2}O_{2}\overset{+v}{\to }O_{2}+2e^{-}+2H^{+}$$

**Figure 1.**
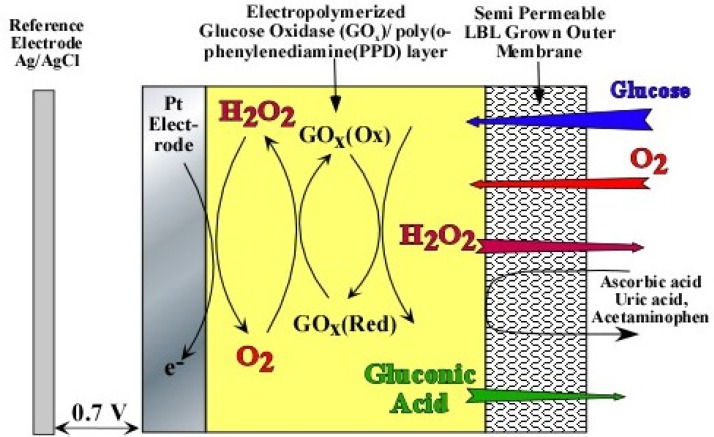
Cross-sectional schematic representation of the multi-layer amperometric glucose sensor used in the numerical simulations. The outer LBL membrane is represented as either one of the following systems: (i) HAs/Fe^3+^ (ii) PSS/PDDA.

As evident from Equations (1) and (2), optimum sensor performance can only be obtained when the glucose to oxygen ratio is less than 1. If not, Equation (2) becomes oxygen limited, which is the case for most *in vivo* applications [8]. For example, in the subcutaneous tissue the O_2_ concentration is only 0.18 mM as compared to 5.6 mM of physiological glucose concentration (glucose/O_2_ ratio ≌ 30) 9], which leads to signal saturation at higher glucose concentrations. In order to mitigate signal saturation and render the sensor response linear, perm-selective membranes based on pore size has been employed, which decrease the glucose to O_2_ ratio in the vicinity of GO_x_ (Figure 1). However, the use of outer membranes inadvertently decreases sensitivity and increases response time.

Second and third generation Clark-type biosensors employ redox mediators and direct ‘wiring’ of enzymes to electrodes in an attempt to minimize or completely eliminate the need for O_2_ [9]. However, the unwanted toxicity associated with the nanomaterials used in second and third generation Clark type glucose sensors stand against their application in implantable sensor configurations [9]. With this in mind, our group has been evaluating mediator-free, 1st generation Clark-type sensors that solely rely on careful mass transfer balance of the various species involved in the operation of these devices [10–13]. As part of this, using a series of layer-by-layer assembled glucose flux limiting membranes (namely humic acids/ferric cations (HAs/Fe^3+^) and poly(styrene sulfonate)/poly(diallyldimethylammonium chloride) (PSS/PDDA)), we have shown that the outer diffusion of H_2_O_2_ through these membranes plays a vital role in determining the sensitivity of the sensor. It was proven that sensors fabricated with highly glucose permeable HAs/Fe^3+^ membranes exhibited lower sensitivities and better linearities compared to sensors with less permeable PSS/PDDA membranes [10]. Through a series of analyte diffusion studies and oxygen dependence of these sensors [10], it was proven that this unique phenomenon did not occur as a result of the poor diffusion of oxygen through the outer membranes, but rather as a result of the outer diffusion of H_2_O_2_ through the various outer layers. In light of these findings, we have shown that optimal sensor performance could be easily achieved via a careful stratification of five functional layers, each of which is intended to carefully balance the mass transfer of various participating species involved [11]. This has also prompted us to theoretically understand the diffusion profiles of various participating species as they diffuse through the polymer and enzyme layers, which is a powerful tool useful in facilitating the development of future high performance first generation Clark-based amperometric implantable sensors.

Herein, we present our initial results from our theoretical analysis of glucose sensors employing the two aforementioned LBL membranes. A multi-layer analyte diffusion model based on Fick's second law was employed to analyze the diffusion profiles of the participating species (namely, glucose and H_2_O_2_). The computational model presented here utilizes well-developed enzyme-catalyzed reaction diffusion equations, which were applied to glucose and H_2_O_2_, to model the reaction of glucose with glucose oxidase. This along with Fick's second law of diffusion, which represents the diffusive transport of glucose and the enzyme-generated H_2_O_2_ throughout the LBL assembled outer membranes, gives a full dynamic representation of the sensor-system under study. The explicit finite difference scheme was coded in MATLAB to solve the diffusion-based partial differential equations in order to obtain concentration profiles of glucose and H_2_O_2_ as a function of LBL thickness and enzyme thickness. In doing this, we were able to precisely analyze the role of H_2_O_2_ in sensor operation and its effect on the amperometric response. In accordance with our previously reported experimental studies [10], sensors coated with the less glucose permeable PSS/PDDA membrane showed a pronounced build-up of H_2_O_2_ compared to that of sensors coated with the more permeable HAs/Fe^3+^ membrane. It was also found that this phenomenon appears for increasing enzyme thicknesses, further confirming the previous experimental studies. Furthermore, amperometric response times were found to increase with increasing enzyme thickness, due to the time necessary for the participating species to establish equilibrium within the enzyme layer.

## 2. The Theoretical Model

Figure 1 shows the cross-section of the sensor under study. The platinum electrode is covered with an electropolymerized GO_x_/poly(*o*-phenylenediamine) (PPD) layer followed by a layer-by-layer assembled outer membrane based on HAs/Fe^3+^ (Device A) and PSS/PDDA (Device B). As can be seen in Figure 1, the incorporation of GO_x_ into the electropolymerized poly(*o*-phenylenediamine) (PPD) has been previously shown to block the oxidation of other endogenous species like uric acid, ascorbic acid and acetaminophen, which are highly likely to oxidize at the operating potential of the sensor [14–16]. The dynamic operation of this multi-layer sensor can be accurately modeled by one-substrate Michaelis-Menten enzyme kinetics [17–21]. First, consider the enzyme-catalyzed reaction where the substrate, S_E_, is irreversibly converted to a product, P_E_:

(4) $$\text{SE}+\text{E}\overset{{\text{k}}_{1}}{\underset{{\text{k}}_{-1}}{⇄}}{\text{ES}}_{\text{E}}\overset{{\text{k}}_{2}}{\to }\text{E}+{\text{P}}_{\text{E}}$$

Here, the substrate, S_E_, is the glucose present in the enzyme layer, the product, P_E_, is the hydrogen peroxide (H_2_O_2_) in the enzyme layer generated per Equation (2), and k_1_, k_−1_ and k_2_ are the rate constants. The rate at which the product is formed, V, can be expressed as:

(5) $$V=\frac{d[{\text{P}}_{\text{E}}]}{dt}=k_{2}[E{\text{S}}_{\text{E}}]$$

**Figure 2.**
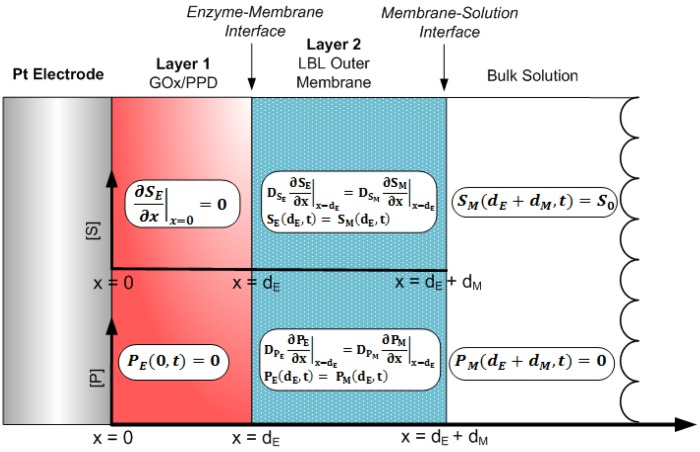
Graphical representation of the Product (H_2_O_2_), P, and Substrate (Glucose), S, boundary conditions employed in the simulated multi-layered glucose sensor. Here, x = 0 is taken at the surface of the Pt electrode, x = d_E_ is taken as the enzyme-membrane interface and x = d_E_ +d_M_ is taken as the surface of the LBL outer membrane.

Applying the quasi steady-state assumption on the rate of variation of the [ES_E_] complex [17] leads to the following equation:

(6) $$V=\frac{d[{\text{P}}_{\text{E}}]}{dt}=\frac{V_{M}[{\text{S}}_{\text{E}}]}{V_{M}+[{\text{S}}_{\text{E}}]}$$

where K_M_ is the Michaelis-Menten constant and V_M_ is the maximum enzymatic rate when the enzyme is fully saturated. We can integrate this rate equation to the well-known diffusion equation to obtain expressions for the effect of enzyme-catalyzed reactions on both glucose and H_2_O_2_ as they diffuse through the GO_x_/PPD layer (layer 1). Here we can safely assume that the GO_x_ is uniformly distributed throughout the layer [22], which is a self-limiting electropolymerization process that yields a thickness comparable to the size of the immobilized enzyme [23]. Additionally, we assume that oxygen is present in sufficient amounts to drive the enzematic reaction to completion. The results in two reaction-diffusion equations (Equations (7) and (8)):

(7) $$\frac{\partial S_{E}}{\partial t}=D_{S_{E}}\frac{\partial^{2}S_{E}}{\partial x^{2}}-\frac{V_{\text{max}}S_{E}}{K_{M}+S_{E}},0<x<d_{E},0<t\leq T$$

(8) $$\frac{\partial P_{E}}{\partial t}=D_{P_{E}}\frac{\partial^{2}P_{E}}{\partial x^{2}}-\frac{V_{\text{max}}S_{E}}{K_{M}+S_{E}},0<x<d_{E},0<t\leq T$$

where *S_E_* (*x*, *t*) and *P_E_* (*x*, *t*) are the concentrations of glucose and H_2_O_2_, respectively, *D_SE_* the diffusion coefficient of glucose in the GO_x_/PPD layer, *D_PE_* the diffusion coefficient of the H_2_O_2_ in the GO_x_/PPD layer, and *d_E_* is the total thickness of the GO_x_/PPD layer. Here, *x* and *t* represent the points in space and time, respectively, where *x* = *0* is taken at the electrode surface, and *x* = *d_E_* at the edge of the GO_x_/PPD layer, and *T* is the total time elapsed. A graphical representation of the boundary conditions of this system conditions can be seen in Figure 2.

The operation of the system initiates immediately after the substrate in the bulk solution reaches the surface of the outer membrane (layer 2), which we define as the following initial conditions:

(9) $$S_{E}(x,0)=0,P_{E}(x,0)=0,x\in [0,d_{E}]$$

(10a) $$P_{M}(x,0)=0x\in [d_{E},d_{E}+d_{M}]$$

(10b) $$S_{M}(dE,0)=0S_{M}(dE+dM,0)=S_{0}$$

Here, we introduce *S_M_* (*x*, *t*) and *P_M_* (*x*, *t*) which are the concentrations of glucose and H_2_O_2_, respectively, in the outer LBL membrane, where *x* = *d_M_* represents the surface of the outer membrane. The assumptions made are the following: (1) the substrate does not react at the electrode surface, (2) the H_2_O_2_ at the electrode surface gets permanently reduced to zero, (3) if the substrate is well stirred, then the diffusion layer (0 < x < *d_E_* +*d_M_*) remains at a constant thickness, *d_E_* + *d_M_*, during sensor operation and (4) the concentration of H_2_O_2_ at the sensor/bulk solution interface is zero. This can be quantitatively described as the following boundary conditions:

(11) $${\frac{\partial S_{E}}{\partial x}|}_{x=0}=0$$

(12) $$S_{M}(d_{E}+d_{M},t)=S_{0}$$

(13) $$P_{E}(0,t)=P_{E}(d_{E}+d_{M},t)=0$$

The aforementioned initial conditions and boundary conditions have been previously employed to model various amperometric and potentiometric biosensors, with various physical parameters and testing conditions [18–21,24–31]. At the boundary between the enzyme layer and the flux-limiting membrane, matching conditions are defined (for *t* > 0) [32]:

(14) $$D_{S_{E}}{\frac{\partial S_{E}}{\partial x}|}_{x=d_{E}}=D_{S_{M}}{\frac{\partial S_{M}}{\partial x}|}_{x=d_{E}},S_{E}(d_{E},t)=S_{M}(d_{E},t)$$

(15) $$D_{P_{E}}{\frac{\partial P_{E}}{\partial x}|}_{x=d_{E}}=D_{P_{M}}{\frac{\partial P_{M}}{\partial x}|}_{x=d_{E}},P_{E}(d_{E},t)=P_{M}(d_{E},t)$$

Following H_2_O_2_ oxidation at the Pt surface, the amperometric current response of the device is expressed as:

(16) $$I=n_{e}D_{PE}F{\frac{\partial P_{E}}{\partial x}|}_{x=0}$$

where, n_e_ is the number of electrons produced (2 for H_2_O_2_), D_PE_ the diffusion coefficient of the H_2_O_2_ in the GO_x_/PPD layer and F is Faraday's constant (96,500 C/mol).

### 2.2. The Solution Scheme

The cross-sectional schematic of Figure 2 was modeled by constructing a mesh employing a finite set of *x* and *t*, and utilizing the boundary conditions of Equations (11) to (15) as a means to obtain a solution to the tridiagonal system. Based on this, the explicit finite difference scheme was used to obtain substrate and product concentrations of the Michaelis-Menten reaction-based differential Equations (7) and (8), as:

***Glucose (S)***

(17) $$S_{E_{i}}^{j+1}=S_{E_{i}}^{j}+\frac{D_{SE}\Delta t}{\Delta x^{2}}(S_{E_{i+1}}^{j}-2S_{E_{i}}^{j}+S_{E_{i-1}}^{j})-\Delta t\frac{V_{\text{MAX}}S_{E_{i}}^{j}}{K_{M}+S_{E_{i}}^{j}}$$

***H****_2_****O****_2_* ***(P)***

(18) $$P_{E_{i}}^{j+1}=P_{E_{i}}^{j}+\frac{D_{PE}\Delta t}{\Delta x^{2}}(P_{E_{i+1}}^{j}-2P_{E_{i}}^{j}+P_{E_{i-1}}^{j})+\Delta t\frac{V_{\text{MAX}}S_{E_{i}}^{j}}{K_{M}+S_{E_{i}}^{j}}$$

where *j* = 1 … *Numt*, where *Numt* represents the total number of time steps and *i* = 1 … *k* − 1, where *k* represents the number of steps in space from the electrode surface to the enzyme/LBL interface. Having said this, at the interface *(i* = *k)* Equations (14) and (15) can be solved explicitly [32] as follows:

***Glucose (S)***

(19) $$S_{i}^{j+1}=S_{i}^{j}+\frac{\Delta t}{\Delta x^{2}}[D_{SM}S_{i+1}^{j}-(D_{SM}+D_{SE})S_{i}^{j}+D_{SE}S_{i-1}^{j}]$$

***H****_2_****O****_2_* ***(P)***

(20) $$P_{i}^{j+1}=P_{i}^{j}+\frac{\Delta t}{\Delta x^{2}}[D_{PM}P_{i+1}^{j}-(D_{PM}+D_{PE})P_{i}^{j}+D_{PM}P_{i-1}^{j}]$$

where *i* = *k* – 1 … *k* + 1. Furthermore, similar to the difference scheme of Equations (17) and (18) and neglecting the Michaelis-Menten reaction portion, the diffusion of glucose and H_2_O_2_ from the bulk through the LBL membrane follows the diffusion equation and was solved as,:

***Glucose (S)***

(17) $$S_{M_{i}}^{j+1}=S_{M_{i}}^{j}+\frac{D_{SM}\Delta t}{\Delta x^{2}}(S_{M_{i+1}}^{j}-2S_{M_{i}}^{j}+S_{M_{i-1}}^{j})$$

***H****_2_****O****_2_* ***(P)***

(18) $$P_{M_{i}}^{j+1}=P_{M_{i}}^{j}+\frac{D_{PM}\Delta t}{\Delta x^{2}}(P_{M_{i+1}}^{j}-2P_{M_{i}}^{j}+P_{M_{i-1}}^{j})$$

where *i* = *k* + 1 … *Numx*, where *Numx* represents the total number of time steps performed in the simulation.

Numerical parameters for the enzymatic layer, such as diffusion coefficients, Michaelis-Menten constant, maximum rate of velocity and thickness have been obtained from earlier reports [22,33]. A keen understanding of the kinetics of H_2_O_2_ diffusion though this multi-layer model has been simulated and analyzed by studying the effect of enzyme thickness (15, 20 and 25 nm GO_x_/PPD) as a function of these two LBL outer membranes. A summation of variables employed in the model can be seen in Table 1.

The physical parameters for the enzyme layer, as well the parameters of the two outer membranes are summarized in Table 2.

## 3. Results

It is well believed that the amperometric response of first generation Clark's glucose sensors is directly proportional to the glucose permeability through the outer membranes assuming that O_2_ is not the limiting factor for the GO_x_ reaction (Equation (2)). Having said this, in order to keep the glucose-to-oxygen ratio less than 1, a variety of outer membranes with less and less glucose permeability have been reported [8,9], albeit without a mechanistic understanding of the diffusion profiles of other participating species such as H_2_O_2_. Layer-by-layer assembled outer membranes afford such studies in that the thickness as well as analyte permeability can be precisely controlled via the number of bilayers within the membrane. Utilizing a series of such LBL assembled membranes (namely a more glucose permeable HAs/Fe^3+^ and less glucose permeable PSS/PDDA membrane), we have previously deduced that the amperometric response of a sensor is also dependent on the outward diffusion of H_2_O_2_. Intuitively, the more glucose-permeable HAs/Fe^3+^ outer membrane is expected to yield a higher response; yet the outwards diffusion of H_2_O_2_ through these membranes is also significant thereby contributing to a lowering of the amperometric response. In addition, through a series of oxygen dependence studies it was concluded that any potential role of lower oxygen levels within HAs/Fe^3+^ membranes in lowering the amperometric response of HAs/Fe^3+^ device has been excluded.

In this contribution, we report a simulation on the concentration profiles of both glucose and H_2_O_2_ throughout the multi-layer system in an attempt to quantify their peak concentrations within the interior of the sensor and to re-assert our earlier finding that H_2_O_2_ is a key contributor in advanced implantable sensor design. Here, it is worth noting that the theory and mathematical analysis of enzyme-catalyzed reactions used in this model are widely-accepted, and thus have been previously applied to similar glucose oxidase systems along with a wide-range of other enzymatic processes. More importantly, based on our earlier experimental studies on oxygen dependence of implantable glucose sensors, we have in this study assumed that O_2_ is present in sufficient amounts (for Equation (2) to go to completion) and that there is no variation in oxygen levels during sensor operation. Moreover, any effect of oxygen within the electropolymerized PPD/GOx layer can be considered negligible based on the fact that the pore size of PPD is enough to let H_2_O_2_ pass through, which is a bigger molecule than O_2_. In fact, it has been reported that sensors having PPD/GO_x_ structures are minimally affected by oxygen [34], reinforcing our O_2_-independnce assumption. Having said this, it should be clear that the assumption of abundant O_2_ levels within the sensor is an approximation, and has been used in order to solely analyze the role of H_2_O_2_ in these devices.

Based on that, we have utilized a one-substrate Michaelis-Menten model [18] in conjunction with diffusion kinetics through the LBL assembled outer membranes to get a holistic representation of sensor performance for the comparison of both devices.

**Figure 3.**
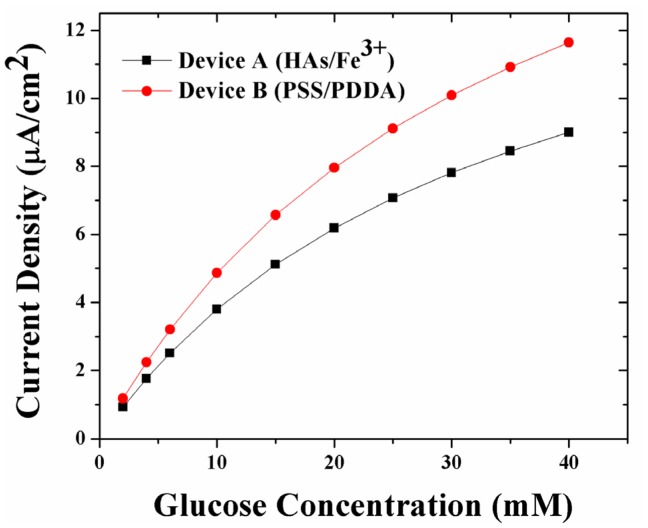
Simulated amperometric response of Device A (100 nm HAs/Fe^3+^) and Device B (5 nm PSS/PDDA) employing 20 nm GO_x_/PPD coating the electrode surface.

Figure 3 shows the simulated amperometric response of sensors coated with the two LBL systems. For any glucose concentration, and in particular at higher glucose concentrations, the amperometric response of Device B (with PSS/PDDA membranes) is 27% higher than that of Device A (HAs/Fe^3+^ membranes). This trend in amperometric response is opposite to the trends observed in glucose permeability through the LBL membranes (which is highest through the HAs/Fe^3+^, Device A, and least through the PSS/PDDA, Device B, membranes), wherein the amperometric response from Device A is 27–30% lower than Device B. Here, it is worth noting that the trend in simulated amperometric responses is similar to that of experimental data reported earlier [10]. Taking into account the low oxygen permeability of PSS/PDDA membranes compared to HAs/Fe^3+^ membranes, it was concluded that the observed higher amperometric response from Device B (PSS/PDDA) is due to a higher build-up of H_2_O_2_ within its sensor geometry. The higher build-up of H_2_O_2_ is in turn attributed to its low permeability through the PSS/PDDA membrane compared to the HAs/Fe^3+^ membrane. In order to further elucidate this, a theoretical understanding of the concentration profiles of glucose and H_2_O_2_ as a function of various LBL membranes has been performed.

**Figure 4.**
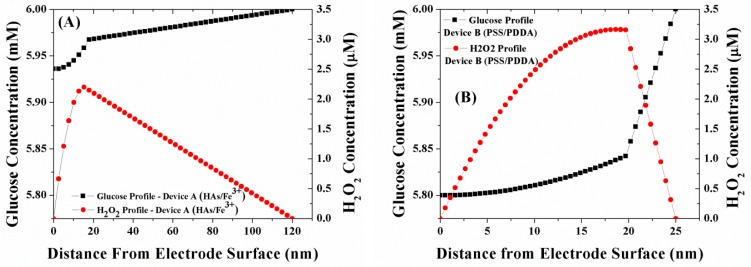
(**A**) Simulated glucose and H_2_O_2_ concentration profiles in the multi-layer sensor system consisting of 20 nm GO_x_/PPD as the first layer, and HAs/Fe^3+^ as the second layer; (**B**) simulated glucose and H_2_O_2_ concentration profiles in the multi-layer sensor system consisting of 20 nm GO_x_/PPD as the first layer, and PSS/PDDA as the second layer.

Figure 4(A,B) illustrates the simulated concentration profile of glucose and H_2_O_2_ within a sensor geometry coated with either of the two LBL membranes namely (HAs/Fe^3+^ and PSS/PDDA) at normal physiological glucose concentrations (6 mM glucose). The GO_x_ enzyme for both devices is immobilized within a 20 mM poly(phenylenediamine) (PPD) layer and is assumed to be uniformly distributed within the PPD layer [22]. Employing the outer membrane thickness values of Table 1, the bulk solution of Device A is present at 120 nm from the electrode surface, while the bulk solution is present at 25 nm from the electrode surface for Device B. As illustrated in Figure 4(A,B), the glucose flux within the GO_x_ layer for Device B (PSS/PDDA) lower than that in Device A (HAs/Fe^3+^), which is in agreement with the trends in glucose permeability through these membranes [12]. Based on the observed lower glucose flux within Device B, one would expect it to have a lower concentration of H_2_O_2_ generated compared to Device A. However, as seen in Figure 4, the concentration of H_2_O_2_ within Device B is 48% higher than that of Device A. Considering the facts that the rate of oxidation of H_2_O_2_ at the Pt electrode is the same for all the electrodes and that the oxygen permeability through the three membranes follows the same trend as glucose [10], the higher concentration of H_2_O_2_ within Device B can easily be attributed to the poor outwards diffusion of H_2_O_2_ through the PSS/PDDA membrane compared to the HAs/Fe^3+^ membrane. This larger build-up of H_2_O_2_ within Device B in turn leads to its higher amperometric response (27–30%) as illustrated in Figure 3.

**Figure 5.**
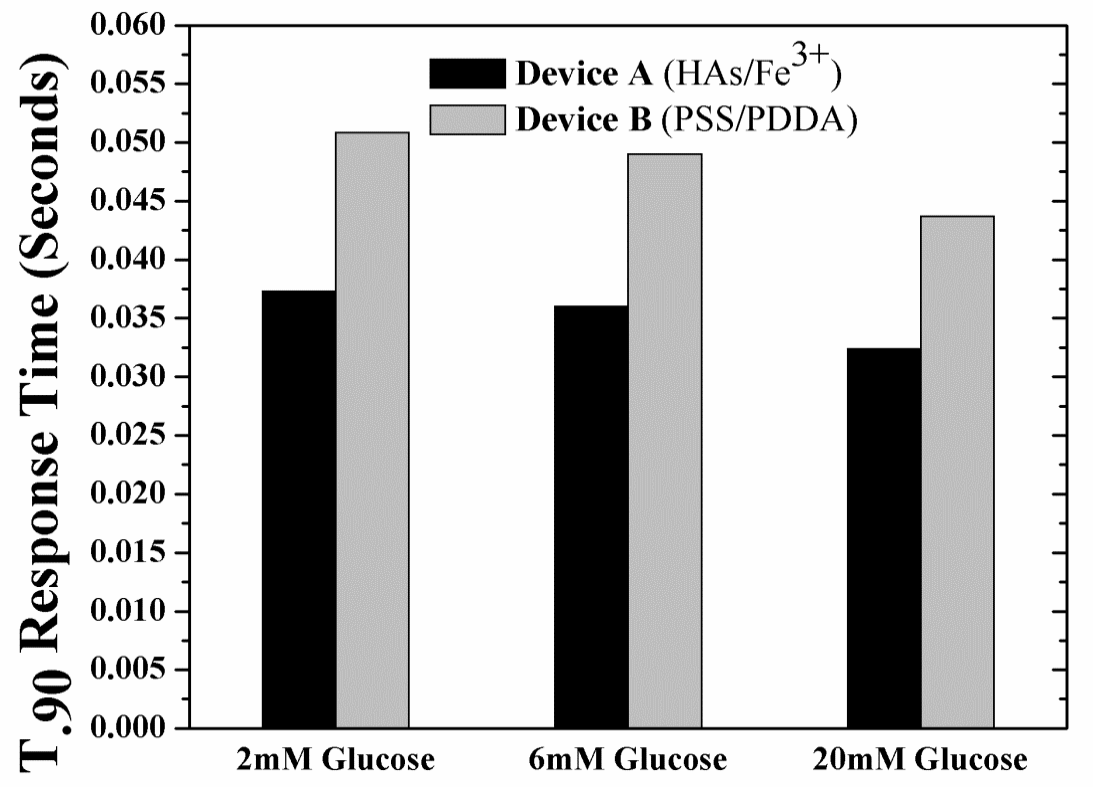
90% saturation current response time *vs.* glucose concentration of Device A and B, employing a 20 nm thick GO_x_/PPD layer.

The unwanted build-up of H_2_O_2_ within Device B is also expected to increase the sensor response time considering the longer times needed to establish equilibrium. This increase in response times adds to the already longer response times resulting from the use of tighter (less glucose permeable membranes). With this in mind, the response times (t_90%_; time taken to reach 90% of the saturation current value as obtained from the simulated sensor transient response, Figure S3 of Supplementary Information) of the devices have been investigated as a function of glucose concentration and type of outer LBL membrane. Figure 5 shows the t_90%_ response times for the two devices under hypoglycemia (2 mM glucose), normal physiological glucose concentrations (6 mM glucose) and hyperglycemia (20 mM glucose). For both devices at any glucose concentration, the response time of Device A was *ca.* 35% less than that of Device B, as expected.

Overall the results of Figures 4 and 5 indicate one has to utilize a tighter PSS/PDDA membrane for higher sensitivities (e.g., for glutamate detection which are in the sub-μM levels [35]) even though it can lead to longer response times (due to build-up of H_2_O_2_) necessitating a means to remove H_2_O_2_ buildup within the sensor. This can be achieved by methods including: (i) increasing the catalytic activity of the Pt working electrode [22] (ii) introducing an additional H_2_O_2_-consuming layer (e.g., Catalase enzyme) [11], and (ii) grading the GO_x_ concentrations in the PPD membrane. Such schemes retain the outer boundary conditions of the currently utilized model (Figure 2), however the inner boundary conditions must be modified in order to include the associated diffusion and reaction-diffusion equations.

**Figure 6.**
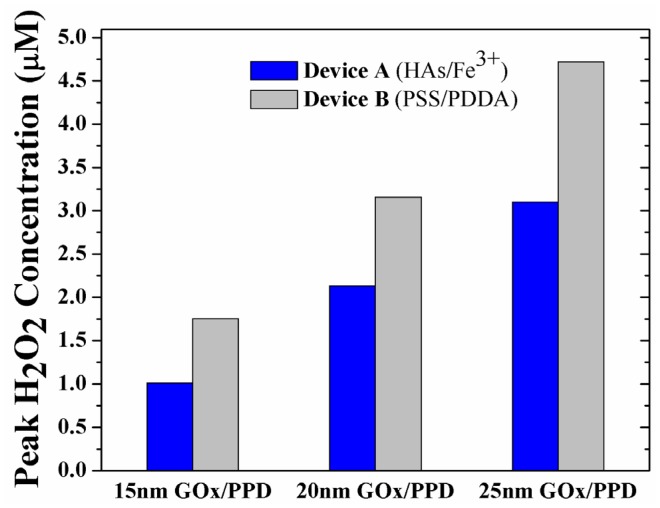
Peak H_2_O_2_ concentration of Device A and B in the GO_x_/PPD layer as a function of enzyme thickness (15, 20 and 25 nm).

Having established the importance of outwards diffusion of H_2_O_2_ on sensor performance, the enzyme layer thickness was additionally simulated at 15 nm and 25 nm, in an effort to determine if the observed phenomenon is enzyme-thickness dependent. Figure 6 shows the peak H_2_O_2_ concentration in the GO_x_/PPD layer of Device A and B at 15 nm (Figure S1 of Supplementary Information), 20 nm (Figure 4) and 25 nm (Figure S2 of Supplementary Information) thick enzyme layers. Intuitively, it is seen that there is increase in peak H_2_O_2_ concentration at increasing enzyme thicknesses, due to the increase in GO_x_-induced H_2_O_2_ turnover. Moreover, for any enzyme thickness, the H_2_O_2_ concentration for Device B is *ca.* 50–70% larger than Device A due to the pronounced buildup of H_2_O_2_ in the enzyme layer.

**Figure 7.**
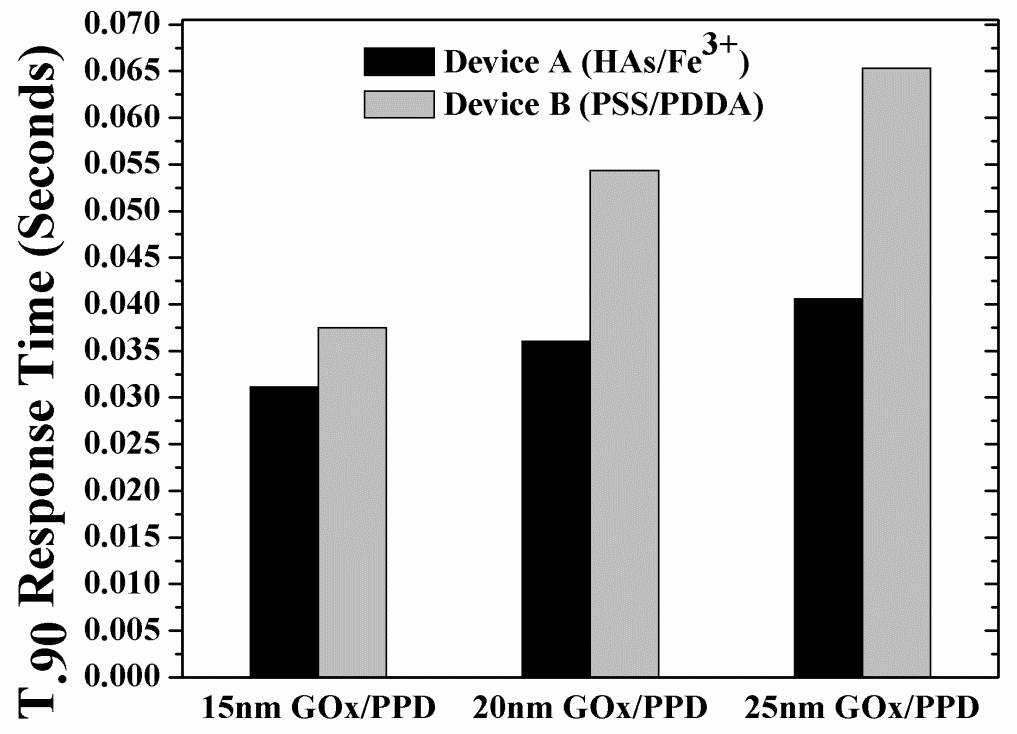
90% saturation current response time *vs.* glucose concentration of Device A and B, as a function of GO_x_/PPD thickness (15, 20 and 25 nm).

Following the analysis of peak H_2_O_2_ concentration as a function of varying GO_x_/PPD thickness, the t_90%_ response times of these thicknesses was investigated. Figure 7 shows the response times of the devices as a function of enzyme thickness. As expected, for any enzyme thickness, the response times of Devices B is higher than that of Devices A, due to the higher build-up of H_2_O_2_ (shown in Figure 6).

## 4. Conclusions

In this contribution, a multi-layered computational model has been utilized to compare the performance of an enzymatic glucose sensor coated with two kinds of LBL assembled outer membranes, namely humic acids/ferric cations and poly(styrene sulfonate)/poly(diallyldimethylammonium chloride) outer membranes. The diffusion profiles of glucose (S) and H_2_O_2_ (P) have been computed under specified boundary conditions for sensors coated with high glucose permeable (HAs/Fe^3^) and low glucose permeable (PSS/PDDA) membranes. Sensors coated with PSS/PDDA membranes, although have low inwards flux, have displayed high sensitivity due to low outwards diffusion of H_2_O_2_ in all simulated enzyme thicknesses. This trend in simulation corroborates our previous experimental finding of the role H_2_O_2_ on sensor sensitivity [10] and re-signifying the need for outer membrane optimization not only in terms of glucose diffusion, but also for H_2_O_2_ diffusion. Moreover, it is also found that this low outer diffusion of H_2_O_2_ through the less glucose permeable PSS/PDDA membranes leads to longer response times thereby alluding to the necessity for a means to remove H_2_O_2_ buildup within an outer membrane-coated biosensor. Currently, we are investigating the efficacy of this model in a variety of physiological environments as well as understanding the need for gradients, if any. Additionally, even though H_2_O_2_ serves as the key contributor to the dynamics of these devices, the role of H_2_O_2_ in an environment of varying oxygen levels is currently under investigation, and will be the topic of future publications.

The study presented here, even though only two LBL membranes were compared, could be extended to other designs that utilize a different outer membrane or to designs that employ a series of stratified membranes and could be used a guide for optimization of outer layers.

## Supplementary Material



## Figures and Tables

**Table 1.** Compilation of variables used in the multi-layer model.

**Declaration of Variables**			
*S_E_*	Concentration of glucose in the GO_x_/PPD layer	*D_SM_*	Diffusion coefficient of glucose in the various LBL outer membranes
*P_E_*	Concentration of H_2_O_2_ in the GO_x_/PPD layer	*D_PM_*	Diffusion coefficient of H_2_O_2_ in the various outer membranes
*S_M_*	Concentration of glucose in the various LBL outer membranes	*V_MAX_*	Maximum enzymatic rate of reaction
*P_M_*	Concentration of H_2_O_2_ in the various outer membranes	*K_M_*	Michaelis-Menten constant
*d_E_*	Thickness of the GO_x_/PPD layer	*I*	Response current
*d_M_*	Thickness of the various LBL outer membranes	*n_e_*	Number of electrons (2 for H_2_O_2_)
*D_SE_*	Diffusion coefficient of glucose in the GO_x_/PPD layer	*F*	Faraday's Constant (96,500 C/mol)
*D_PE_*	Diffusion coefficient of H_2_O_2_ in the GO_x_/PPD layer		

**Table 2.** Physical parameters for the enzyme layer and LBL assembled outer membrane used in the simulation, including diffusion coefficients for glucose and H_2_O_2_, membrane thicknesses, Michaelis-Menten constant and maximum rate of enzymatic activity.

	**Layer**		
**Parameter**	**GO_x_/PPD**	**HAs/Fe^3+^**	**PSS/PDDA**
*D_Glucose_ (cm^2^/sec)*	4 × 10^−10^ [22,33]	4.4 × 10^−9^ [10]	0.058 × 10^−9^ [10]
*D_H2O2_ (cm^2^/sec)*	5 × 10^−9^ [22,33]	4.8 × 10^−9^ [10]	0.1 × 10^−9^ [10]
*Thickness (nm)*	15, 20 and 25 [33]	100 [10,12]	5 [10,12]
*K_M_ (mol/cm^3^)*	33 × 10^−6^ [22,33]	n/a	n/a
*V_Max_* (*mol/cm^2^sec)*	60 × 10^−6^ [22,33]	n/a	n/a
