# Supplementary material for: Theoretical Analysis of the Performance of Glucose Sensors with Layer-by-Layer Assembled Outer Membranes
Source: Sensors (Basel). 2012 Oct 1;12(10):13402–16. doi: 10.3390/s121013402 (PMC3545572; doi:10.3390/s121013402)

Supplementary Information

Theoretical Analysis of the Performance of Glucose Sensors with Layer-by-Layer Assembled Outer Membranes

Robert A. Croce Jr. [1], **Santhisagar Vaddiraju** [2,3], **Fotios Papadimitrakopoulos** [3,4] **and Faquir C. Jain** [1,*]

[1] Electrical and Computer Engineering, University of Connecticut, 371 Fairfield Way, Storrs, CT 06269, USA; E-Mail: rjc03001@engr.uconn.edu

[2] Biorasis Inc., Technology Incubation Program, University of Connecticut, Storrs, CT 06268, USA

[3] Nanomaterials Optoelectronics Laboratory, Polymer Program, Institute of Materials Science, University of Connecticut, Storrs, CT 06269, USA; E-Mails: sagar@ims.uconn.edu (S.V.); papadim@ims.uconn.edu (F.P.)

[4] Department of Chemistry, University of Connecticut, Storrs, CT 06269, USA

Figures S1 and S2 show the concentration profiles of glucose and H_2O_2 for Devices A and B at a GO_x/PPD thickness of 15 and 25 nm, respectively. For both these GO_x/PPD thicknesses the concentration of H_2O_2 is higher in Device B (PSS/PDDA) compared to Device A (HAs/Fe^{3+}), similar to that shown in Figure 4. This confirms that tighter (less glucose permeable) outer membranes (Device B) leads to higher build-up of H_2O_2 within the sensor geometry and the phenomena is independent of the enzyme thickness. Figures 5 and 7 in the manuscript represent the time taken for the sensor to reach 90% of its saturation value. In order to obtain these values, the transient response of the sensor shown in Figures S3 was utilized. These transient responses were obtained utilizing Equation (16).

Figure S1. (**A**) Simulated glucose concentration profile in the multi-layer sensor system consisting of 15 nm GO_x/PPD as the first layer, and different LBL membranes as the second layer (HAs/Fe^{3+} and PSS/PDDA); (**B**) simulated H_2O_2 concentration profile in the multi-layer sensor system consisting of 15 nm GO_x/PPD as the first layer, and different LBL membranes as the second layer (HAs/Fe^{3+} and PSS/PDDA).

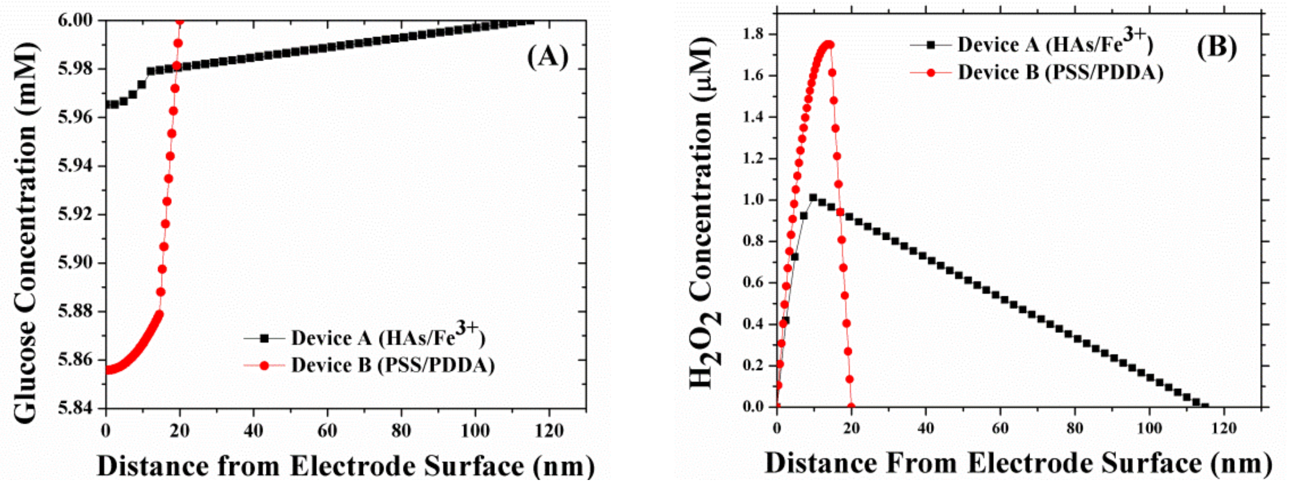

Figure S2. (**A**) Simulated glucose concentration profile in the multi-layer sensor system consisting of 25 nm GO_x/PPD as the first layer, and different LBL membranes as the second layer (HAs/Fe^{3+} and PSS/PDDA); (**B**) simulated H_2O_2 concentration profile in the multi-layer sensor system consisting of 25 nm GO_x/PPD as the first layer, and different LBL membranes as the second layer (HAs/Fe^{3+} and PSS/PDDA).

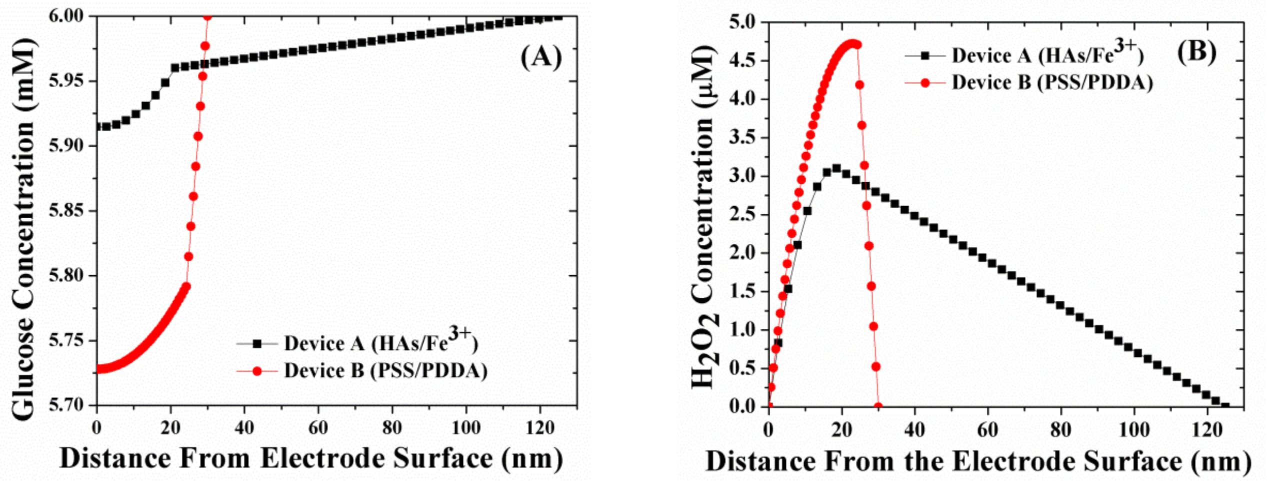

Figure S3. Simulated transient response of Devices A and B at (**A**) 2 mM, (**B**) 4 mM and (**C**) 20 mM glucose.

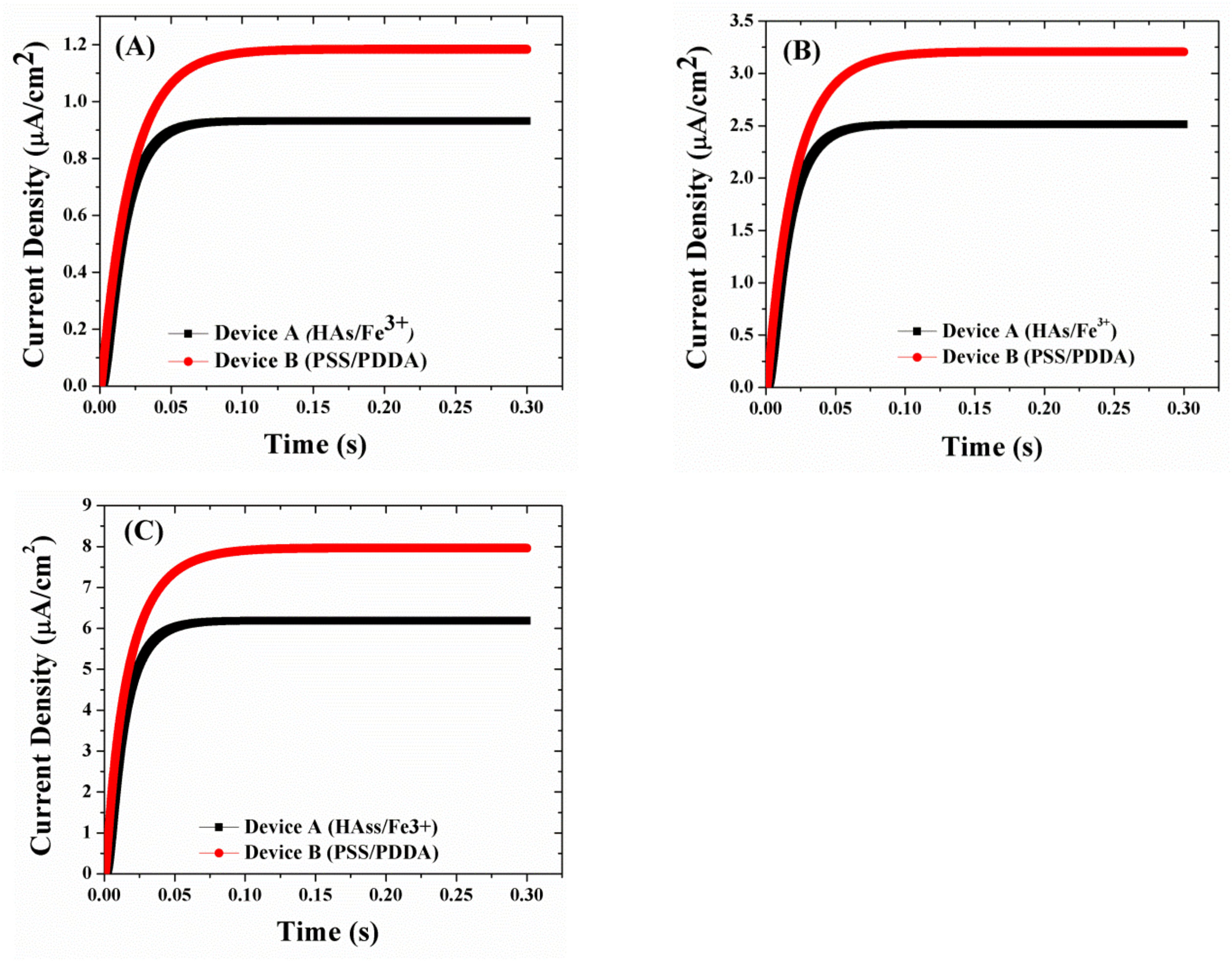

Supplement: Supplementary file 1 [file sensors-12-13402-s001.pdf]
